# Advanced technical tips and recent insights in ERCP using balloon‐assisted endoscopy

**DOI:** 10.1002/deo2.301

**Published:** 2023-11-14

**Authors:** Masaaki Shimatani, Toshiyuki Mitsuyama, Takeshi Yamashina, Masahiro Takeo, Shunsuke Horitani, Natsuko Saito, Hironao Matsumoto, Masahiro Orino, Masataka Kano, Takafumi Yuba, Takuya Takayama, Tatsuya Nakagawa, Shoji Takayama

**Affiliations:** ^1^ Division of Gastroenterology and Hepatology Kansai Medical University Medical Osaka Japan

**Keywords:** double‐balloon endoscopy, ERCP, pancreaticoduodenectomy, Roux‐en‐Y reconstruction, single‐balloon endoscopy

## Abstract

Pancreatobiliary endoscopic interventions using balloon‐assisted endoscopes have been widely acknowledged as the first‐line therapy for pancreatobiliary diseases in postoperative patients with reconstructed gastrointestinal anatomy (excluding the Billroth I procedure). However, there are many technical difficulties, and the procedural completion rates vary in a wide range among institutions, indicating the procedural technique is yet to be standardized. This article aims to provide technical tips of procedures and insights into the advanced aspects, including the management of extremely difficult cases and troubleshooting of endoscopic retrograde cholangiopancreatography using balloon endoscopy, along with a review of recent advancements in this field.

## INTRODUCTION

Since its development in 1968, endoscopic retrograde cholangiopancreatography (ERCP) has undergone continuous advancements and become an indispensable diagnostic and therapeutic modality for pancreatobiliary disorders.^1^, ^2^, ^3^ However, the endoscopic approach for pancreaticobiliary disease with postoperative reconstructed intestines is challenging due to unique anatomical characteristics, such as the distance from the Y‐anastomosis and curvature, as well as the distance to the Y‐anastomosis and postoperative adhesions. Conventional endoscopes often failed to reach targeted sites, such as blind ends, papillae, and hepaticojejunal/pancreaticojejunal anastomosis,^4^, ^5^, ^6^, ^7^ which resulted in unsatisfactory procedural outcomes resorting to percutaneous or surgical interventions. Percutaneous approaches have limited applicability in cases involving bleeding diathesis or ascites, and pancreatic diseases are not applicable. Surgical interventions are considered highly invasive. Therefore, balloon‐assisted endoscopy (BAE), initially developed for the diagnosis and treatment of small intestinal disorders, has been adapted for ERCP (BAE‐assisted ERCP; BAE‐ERCP) to enable endoscopic access. Although numerous reports have reported the usefulness and safety of BAE‐ERCP for the management of biliary diseases,^8^, ^9^, ^10^, ^11^ the procedures remain challenging in many instances and success rates vary in a wide range among facilities.^12^ The standardization of this technique is yet to be established. However, recent studies have reported improved success rates,^13^, ^14^, ^15^, ^16^ and a comprehensive review indicated equivalent pooled endoscopic, diagnostic, and procedural success rates between BAE‐ERCP and conventional ERCP, with rates of 90% (95% confidence interval [CI], 84%–94%), 94% (95% CI, 88%–98%), and 93% (95% CI, 88%–97%), respectively.^17^

Regarding pancreatic diseases, the use of BAE for endoscopic treatment has long been considered impractical, with minimal reports on such interventions. However, recent advancements have introduced novel approaches, effectively establishing BAE‐assisted endoscopic therapies as a feasible option.

This article describes tips and troubleshooting for balloon endoscopic biliopancreatic endoscopic treatment and reviews the latest insights in BAE‐ERCP, along with a review of recent literature.

## BALLOON‐ASSISTED ENDOSCOPY

The BAE is designed on the novel concept of grasping and shortening intestine using a balloon/balloons, allowing scope advancement for deep insertion.^18^ Currently, commercially available BAE systems in Japan include the double‐balloon endoscope (DBE) manufactured by FUJIFILM and the single‐balloon endoscope (SBE) manufactured by Olympus. As specifically designed for biliary and pancreatic endoscopic treatments, FUJIFILM introduced the short‐type DBE (working length: 155 cm, working channel: 3.2 mm; EI‐580BT), and Olympus released the short‐type SBE (working length: 152 cm, working channel: 3.2 mm; SIF‐H290S) in 2016. These dedicated devices for pancreatobiliary endoscopy feature a shorter working length and larger working channel, allowing most of existing devices for pancreatobiliary interventions applicable. Consequently, short‐type BAE has been reported to safely accomplish procedures equivalent to conventional ERCP, making it a useful tool for pancreatobiliary endoscopic treatment.^19^, ^20^, ^21^ Furthermore, in order to improve scope inserting maneuverability in patients with postoperative reconstructed intestines, the short‐type DBE is equipped with an advanced force transmission function and adaptive bending system,^19^, ^20^ whereas the short‐type SBE incorporates high‐force transmission and passive bending system.^21^

Comparative studies evaluating the utility of DBE and SBE have reported no significant differences in success rates and adverse events regardless of the type of scopes used.^22^ It is considered important to understand the characteristic features of respective scopes and use appropriately.

## RECONSTRUCTION PROCEDURES

The most common reconstructive methods for gastrectomy for gastric and duodenal disease are Billroth I, Billroth II, and Roux‐en‐Y reconstructions. There are also double tract and jejunal interposition reconstruction methods that aim at physiological anastomosis; however, with the increase in the number of laparoscopic surgeries in recent years, the Roux‐en‐Y reconstruction method is often used after gastrectomy. Reconstructive methods for pancreaticoduodenectomy (PD) for pancreatic cancer and lower bile duct cancer include the Child (modified), Whipple, and Imanaga methods. Currently, in Japan, the Child (modified) method is primarily applied. For patients with hilar and proximal bile duct cancers, as well as congenital biliary dilatation and biliary malformation, the gastric‐preserving Roux‐en‐Y reconstruction method is commonly performed.

## TECHNICAL REVIEWS OF BAE‐ERCP

### Techniques for deep insertion of endoscope based on reconstruction methods

One of the challenges in achieving successful ERCP using BAE is reaching the target site^23^; however, not all the postoperative cases with reconstructed gastrointestinal anatomy pose technical challenges for endoscope insertion. The reconstruction method does affect the difficulty level^8^, ^16^; however, it should be noted that BAE facilitates endoscopic insertion in most reconstruction methods, with only a few specific methods hindering it. Awareness of these challenging cases and a thorough understanding of the characteristics and the risk associated with each reconstruction method are essential.

#### Billroth II reconstruction

In Billroth II reconstruction, there are two types: the short afferent loop type and the long afferent loop (LAL) type. The LAL type often involves a jejunojejunostomy called the Braun anastomosis between the afferent and efferent limbs. These anatomical variances of reconstruction method affect the difficulty of scope insertion.

In the short afferent loop type, the sharp angle of anastomosis precludes identifying the opening of the presumed afferent limb, and scope maneuver is also hindered. The opening of the afferent limb is usually located upper left of the anastomotic site, though it cannot be observed from the gastric lumen due to the collapsed lumen on the opposite side of the efferent limb. The key to inserting into the afferent limb is to use an upward angle to align the scope with the afferent limb and gradually apply a downward angle while “pulling” the scope, thus aligning the direction of the scope tip with the direction of the afferent limb and sliding it into the afferent limb.

In LAL type, there are usually no significant difficulties in inserting the scope into the afferent limb. Both openings of the intestinal limbs are visible from the gastric lumen, making it relatively easy to insert the scope into either limb. However, cases with a long afferent limb may present challenges due to adhesions. Additionally, in cases where the afferent and efferent limbs are anastomosed using a Braun anastomosis, the lumens can appear like a maze, requiring caution. Nevertheless, the use of Braun anastomosis can facilitate smooth insertion into the blind end.

#### Child (modified) method

In the Child (modified) method, the reconstruction should be similar to the aforementioned LAL type. Two intestinal openings can be seen from within the gastric cavity, and the scope can be inserted into either opening relatively smoothly. In cases where the afferent and efferent limbs are anastomosed by Braun anastomosis, the lumen may appear like a maze, which may seemingly look challenging. However, the Braun anastomosis can be effectively utilized based on proper anatomical comprehension, facilitating scope insertion to the blind end.^24^ In cases of Braun anastomosis, the blind end can be reached regardless of whether the scope is inserted through the afferent or efferent limb route at the gastrojejunostomic site. When inserting through the afferent limb route, advancing to the most distal lumen without crossing the suture line of the Braun anastomosis leads to the blind end. However, when inserting through the efferent limb route, advancing beyond the suture line of the Braun anastomosis and into the central lumen leads to the blind end. In other words, in cases of the Braun anastomosis, inserting through the efferent limb route via the Braun anastomosis facilitates a milder inserting angle toward the blind end, allowing stable endoscopic maneuverability during biliary interventions.^24^

#### Roux‐en‐Y reconstruction

Generally, Roux‐en‐Y reconstruction is considered the most challenging reconstruction method. In the comparison of two cases of gastrectomy (Roux‐en‐Y with total/partial gastrectomy) and gastric preservation (Roux‐en‐Y with hepaticojejunostomy), gastric preservation cases are considered more difficult for scope insertion than gastrectomy cases.

##### Roux‐en‐Y reconstruction after gastrectomy (Roux‐en‐Y with total/partial gastrectomy)

The first concern for Roux‐en‐Y reconstruction is identifying the afferent limb when reaching the Y‐anastomosis. Several reports have described useful methods for identifying the afferent limb.^25^, ^26^

The Y‐anastomosis can be sorted into end‐to‐end or side‐to‐side anastomosis. In addition, in order to identify the afferent limb, an insertion technique using the “suture line” of the Y‐anastomosis should be helpful.^25^ In cases of Roux‐en‐Y with gastrectomy, end‐to‐end anastomosis is generally more common, although side‐to‐side anastomosis can be selected for factors such as instrument‐assisted anastomosis or surgeon preference. In end‐to‐end anastomosis, the afferent limb is sutured on the lateral side of the elevated jejunum, which requires special attention for detection. The blind end should be reached by selecting the lumen on the side beyond the “suture line,” where Y‐anastomosis is split into two limbs. In side‐to‐side anastomosis, three lumens are observed at the Y‐anastomosis, and advancing toward the middle lumen beyond the “suturing line” should lead to the afferent limb. As a technical tip for endoscope insertion, it is important to insert the scope carefully by forming like a loop instead of stretching the scope, and by using angle manipulation rather than pushing. Another tip is to insert the scope forming a shape that facilitates cannulation during biliary approach. In other words, it is necessary to insert with consideration for how to facilitate subsequent ERCP‐related procedures, such as forming a large reverse α‐loop, which would allow positioning the major papilla in a facing direction through the retroflex position.

##### Roux‐en‐Y with preserved stomach (Roux‐en‐Y with hepaticojejunostomy)

Roux‐en‐Y with hepaticojejunostomy is the most challenging method in terms of reaching the blind end due to its anatomical characteristics, including a long distance to the blind end, multiple flexures, susceptibility to scope bending within the stomach, and potential adhesion influences. In this context, it is crucial to stretch the scope as much as possible to maintain its maneuverability, unlike in Roux‐en‐Y with gastrectomy.

The Y‐anastomosis can involve either end‐to‐end or side‐to‐side anastomosis. In the case of side‐to‐side anastomosis, the “suture line” can be used as in the Roux‐en‐Y with the gastrectomy reconstruction method. However, end‐to‐end anastomosis by Roux‐en‐Y with hepaticojejunostomy in contrast to Roux‐en‐Y with gastrectomy takes a bifurcating form beyond the “suture line,” precluding to use the “suture line” for insertion. In the case of end‐to‐end anastomosis, the intraluminal injection of indigo carmine should be effective.^27^ However, even if the afferent limb could be identified, it is still difficult to advance the scope retrogradely toward the blind end due to the sharp flexure of Y‐anastomosis hindering the transmission of the maneuvering force to scope tip. The short‐type DBE has been developed to address this issue and is equipped with an advanced force transmission function and adaptive bending system.^19^, ^20^ Moreover, the short‐type SBE features high‐force transmission and passive bending mechanisms,^21^ both facilitating insertion beyond the Y‐anastomosis into the afferent limb.

**FIGURE 1:**
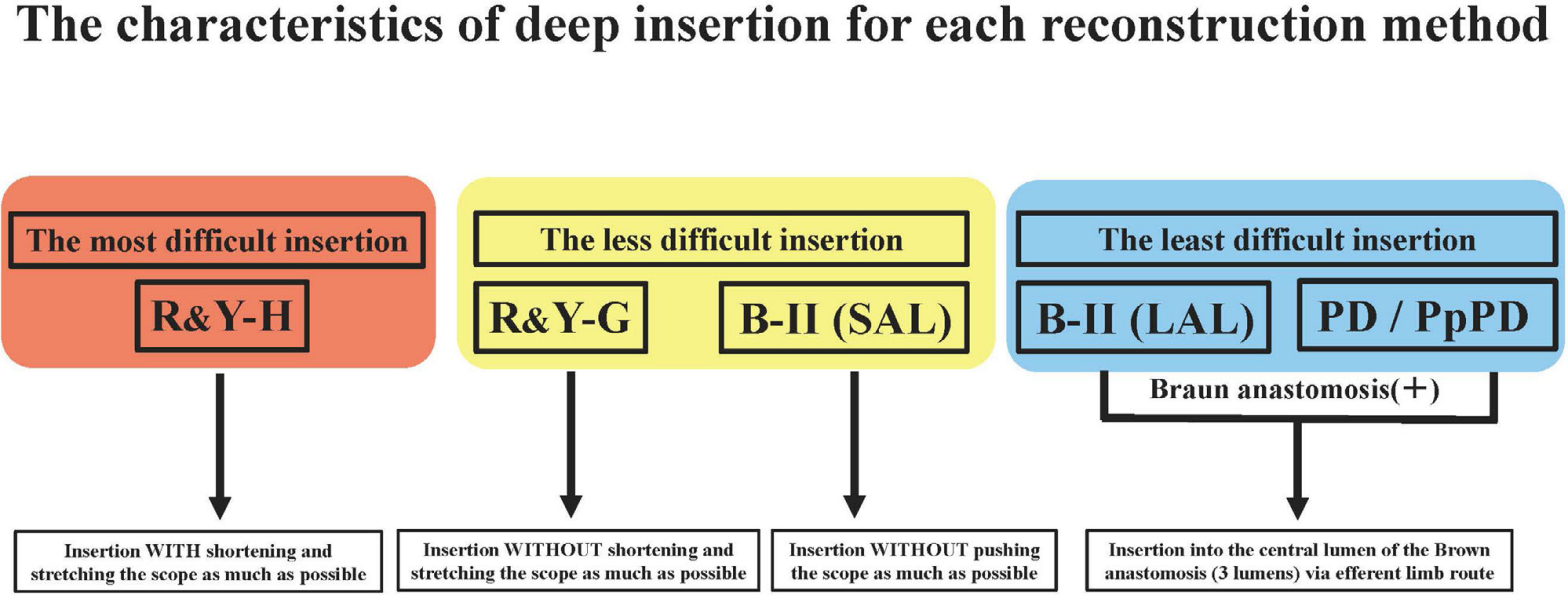
The characteristics of deep insertion for each reconstruction method. Insertion for Roux‐en‐Y with hepaticojejunostomy is the most difficult. The insertion of Roux‐en‐Y with hepaticojejunostomy should be inserted with a shortened scope, whereas Roux‐en‐Y with gastrectomy should be inserted without. Insertion for B‐II (long afferent loop) and pancreaticoduodenectomy/pylorus preserving pancreatoduedenectomy is the least difficult. B‐II, Billroth II gastrectomy; LAL, long afferent loop; PD, pancreaticoduodenectomy; PpPD, pylorus preserving pancreatoduodenectomy; R&Y‐G, Roux‐en‐Y with gastrectomy; R&Y‐H, Roux‐en‐Y with hepaticojejunostomy; SAL, short afferent loop.

In short summary, the difficulty level of scope insertion varies according to the reconstruction methods. The gastric preservation Roux‐en‐Y reconstruction method is considered the most difficult, whereas the Child (modified) method and Billroth II gastrectomy (LAL type) are regarded relatively easier. It is important to be acquainted with the insertion technique for respective reconstruction methods to improve the success rate of endoscopic insertion.^28^, ^29^ The characteristics of deep insertion for each reconstruction method are summarized in Figure 1.

### Technical tips for biliary approaches

The biliary approach includes the transpapillary approach and the transcholedochal jejunal anastomostic approach. It has been reported that the transpapillary approach is more difficult than the transcholedochal jejunal anastomostic approach.^30^ This section provides an overview of the technical tips for each approach.

#### Transpapillary approach

**FIGURE 2:**
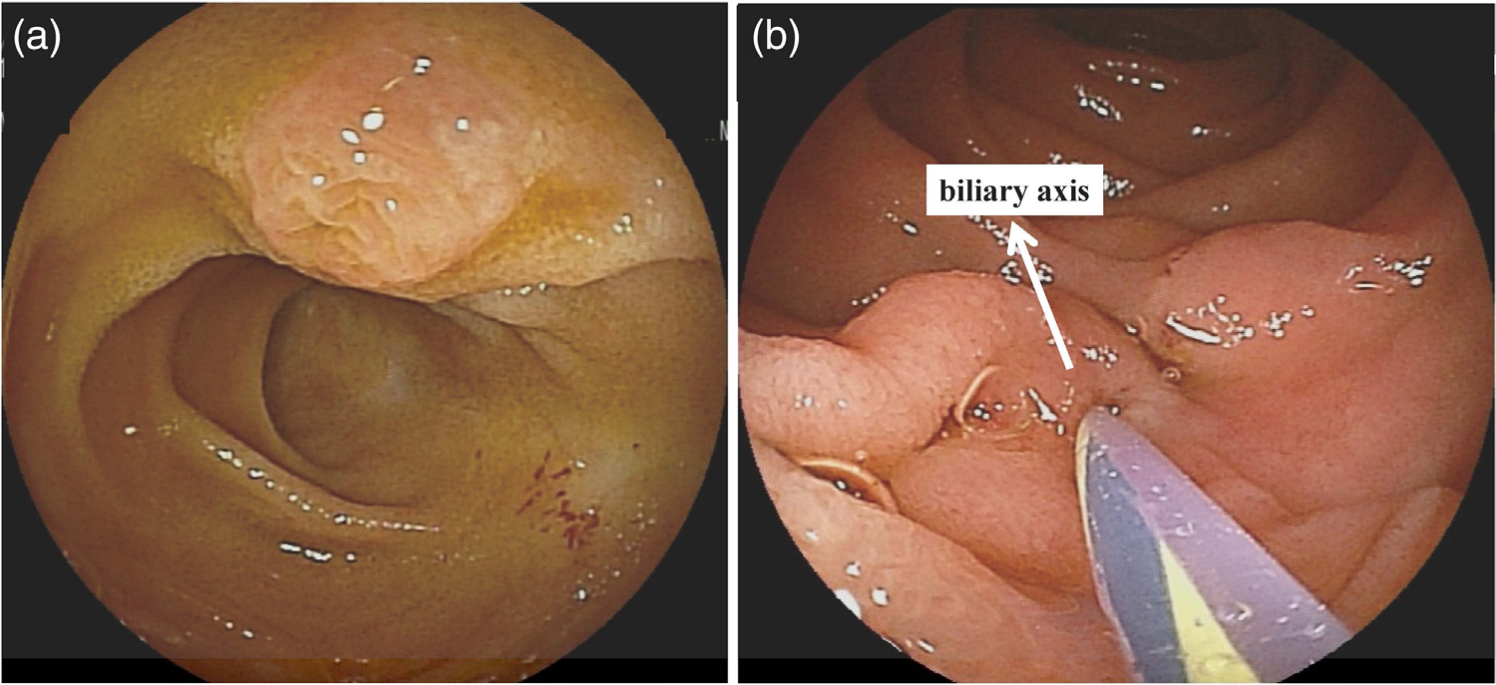
Positioning of major papilla (with double‐balloon endoscope) for Billroth II reconstruction: (a) papilla on reaching the blind end (b). The major papilla was positioned at 6m o'clock direction with working channel in 5:30 direction.

The reconstruction methods for the transpapillary approach include Billroth II reconstruction and Roux‐en‐Y reconstruction with gastrectomy. In Billroth II reconstruction, the papilla is observed on the left or upper side of the screen after reaching the blind end (Figure 2a), which is upside down to the normal ERCP view. What is important here is to release the loop formed by scope as much as possible and grasp the intestine using each balloon on the tip, which should prevent the scope slipping and enable stable maneuverability. However, excessive tension on the intestine can lead to perforation. In order to avoid perforation, the overtube should not be inserted too close to the blind.

**FIGURE 3:**
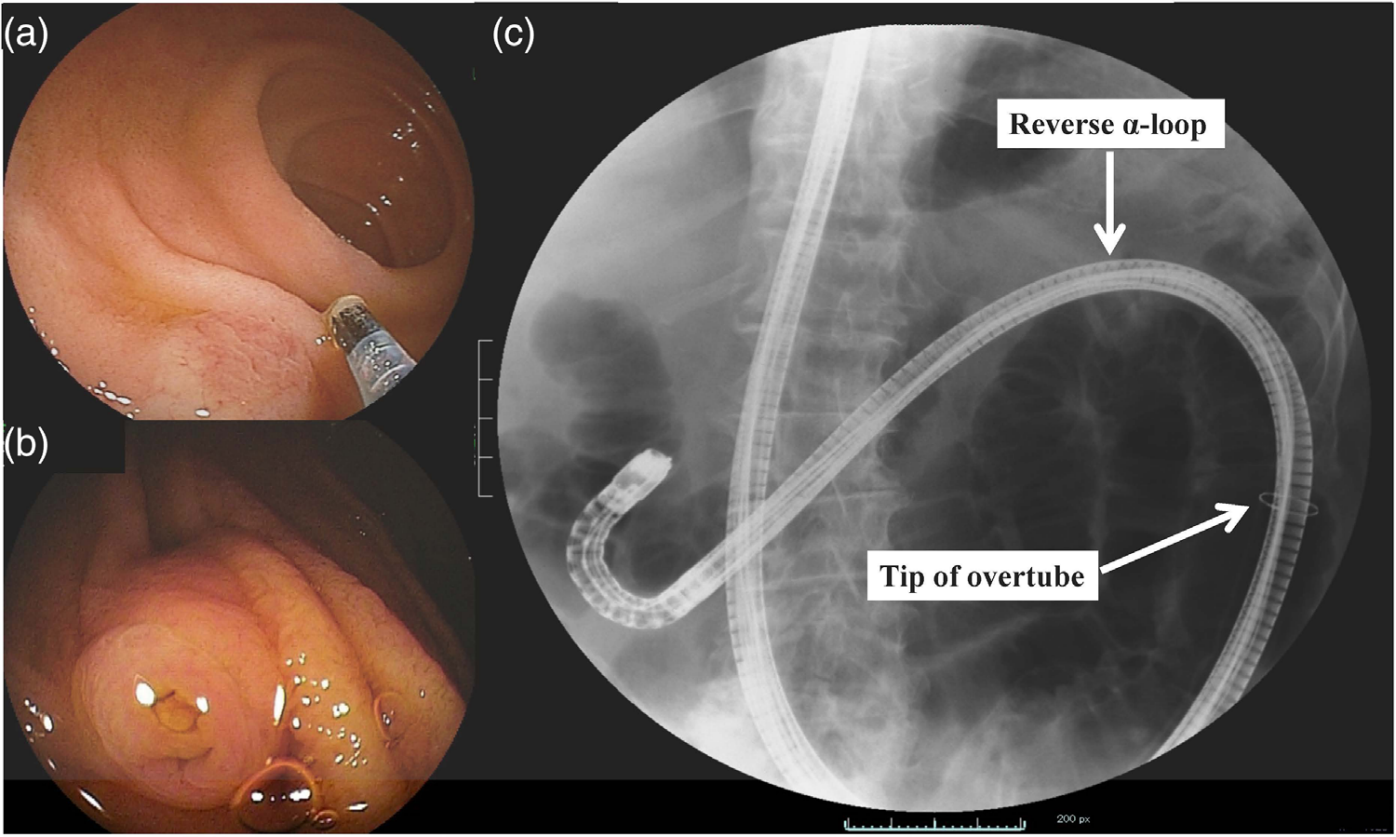
The view of major papilla in Roux‐en‐Y reconstruction (double‐balloon endoscope case): (a) tangential major papilla observation, (b) major papilla observation in retroflex position, and (c) scope shape and overtube position in retroflex position.

On the other hand, in the case of Roux‐en‐Y reconstruction after gastrectomy, the papilla is frequently observed in the 11–1 o'clock direction, in an upward orientation when reaching the blind end. However, it may also be visualized in various locations on the screen, requiring careful search for the major papilla. The important point is that the major papilla is often observed in the direction of the tangential line (Figure 3a), and it is necessary to bring the scope in retroflex position where a facing view of the major papilla through a combination of angle and scope manipulation (Figure 3b). Therefore, the scope loops should intentionally be maintained, forming a large reverse α‐loop, and the overtube should not be advanced too deeply into the intestine but rather halted in the middle of the reverse α‐loop (Figure 3c). Proper positioning of the major papilla during biliary cannulation in Roux‐en‐Y reconstruction after gastrectomy is crucial, and for this purpose, the shape of the scope during insertion is important.^23^

The suitable positioning of the major papilla varies depending on the type of applied scope. In the case of the short‐type DBE, the key to biliary cannulation is to bring the major papilla in the 6 o'clock direction on the screen. With the short‐type DBE, the catheter appears from the 5:30 direction on the endoscopic view. By aligning the papilla in this position, the catheter can be positioned downward in alignment with the biliary axis (Figure 2b). Applying gentle pressure to the major papilla using a straight‐type catheter and delicate axial alignment with the bile duct should facilitates biliary cannulation.

**FIGURE 4:**
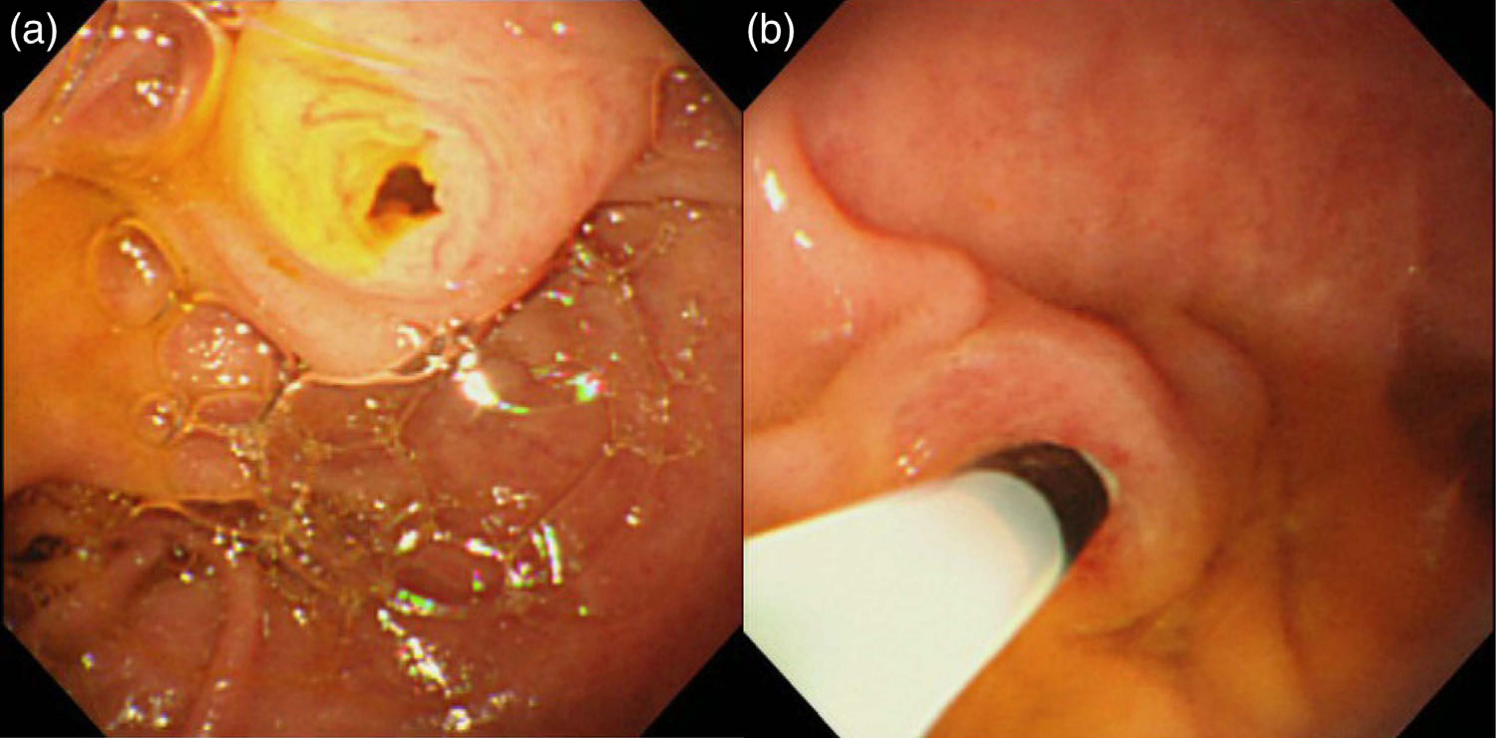
The view of major papilla in Roux‐en‐Y reconstruction (single‐balloon endoscope case): (a) the major papilla was positioned at 11–12 o'clock, and (b) the catheter appears from the 8 o'clock direction on the endoscopic view.

Regarding the short‐type SBE, the catheter appears from the 8 o'clock direction with this endoscope, and the suitable position for the major papilla should be in the 9–11 o'clock direction on the screen (Figure 4a,b).

#### Choledochojejunal anastomostic approach

**FIGURE 5:**
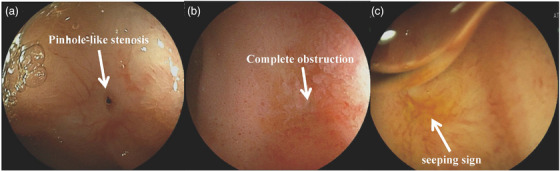
Benign stenosis at the choledocojejunal anastomosis: (a) pinhole‐like stenosis, (b) complete obstruction, (c) "seeping sign" observed underwater.

The reconstruction methods with choledochojejunal anastomostic approach include the Child (modified) method and the Roux‐en‐Y with hepaticojejunostomy method. First, it is important to identify choledochojejunal anastomosis. In the case of a benign stenosis with a pinhole‐like strictured anastomotic site (Figure 5a), special attention is required for detection. The fluoroscopic images can help presume the location of choledochojejunal anastomosis, and careful searching around the anastomosis can help identify any signs of bile outflow. The presence of ulcer scar‐like mucosa surrounding the anastomosis can also serve as a clue for finding the anastomotic site.^31^ In cases where the choledochojejunal anastomosis is completely occluded (Figure 5b), filling the blind end with saline solution and inflating an endoscopic balloon if DBE to open the intestinal folds allows for underwater observation. If there is any visible sign of bile flow in the water (seeping sign) (Figure 5c), then the chance of successful detection of the choledochojejunal anastomosis should be quite high.^32^

### Technical tips for pancreatic approaches

**FIGURE 6:**
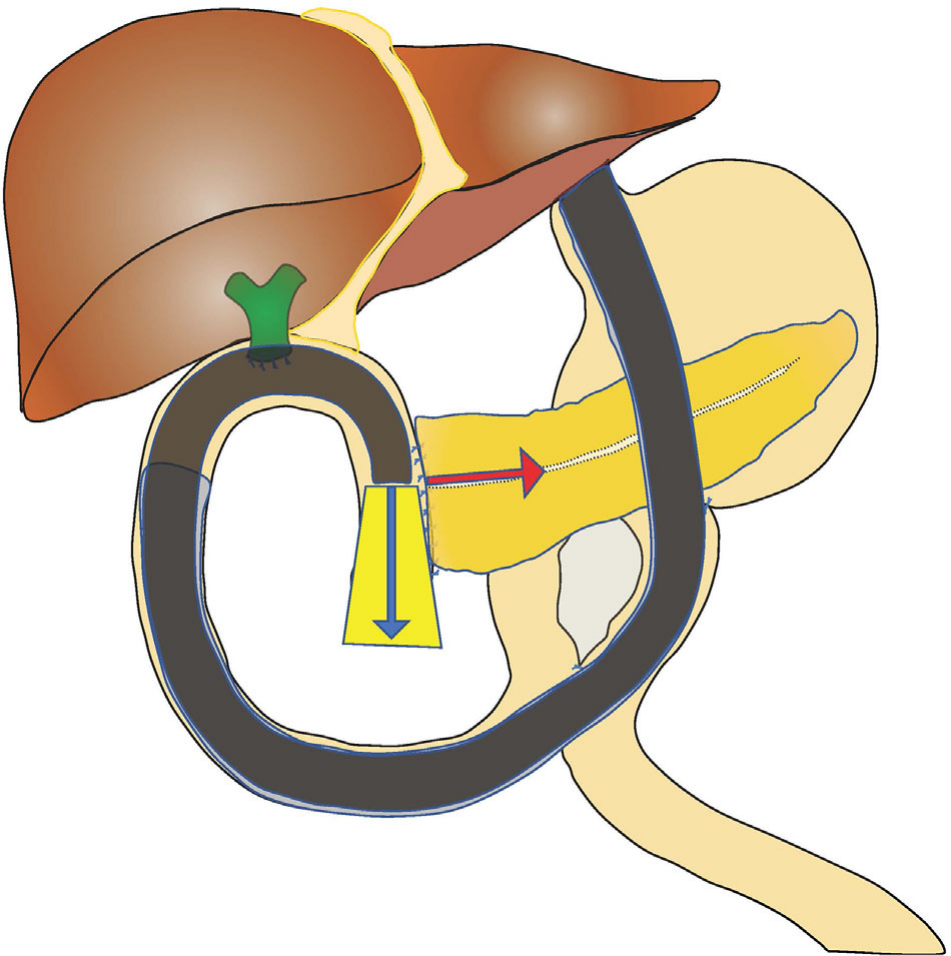
Schema of pancreaticoduodenectomy (PD)‐IIa reconstruction method. Pancreaticojejunal anastomosis (red arrow) is tangential to the endoscopic view (blue arrow).

**FIGURE 7:**
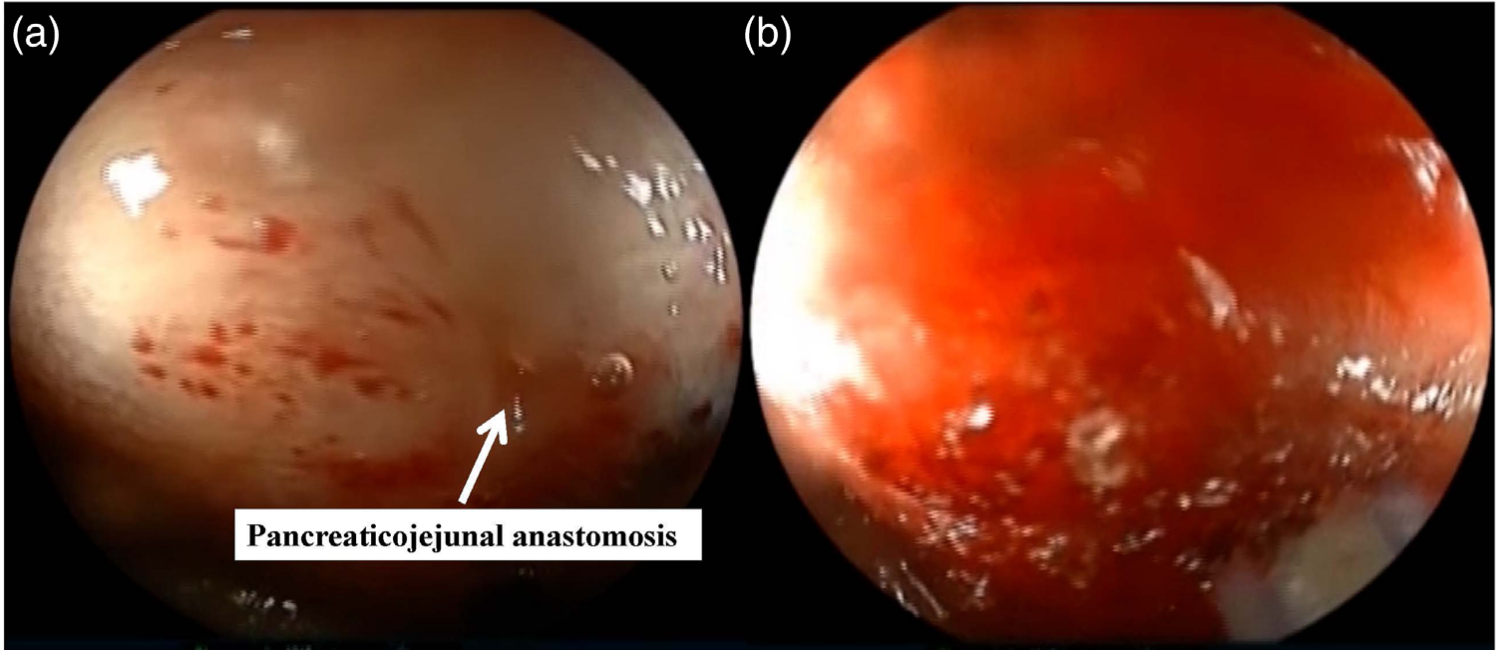
Pancreaticojejunal anastomosis approach using the conventional method: (a) pancreaticojejunal anastomosis (arrow), (b) tends to be a blind approach during pancreatic cannulation.

There are two approaches to the pancreatic duct: the transpapillary approach and the transpancreatico jejunal anastomotic approach. The frequently challenged issue is the approach to the pancreaticojejunal anastomosis after PD with child (modified) procedure, which is the most commonly performed approach in Japan. The reasons the endoscopic approach using BAE is regarded difficult are as follows: (1) The pancreaticojejunal anastomosis is located tangentially to the endoscopic view and hidden by the intestinal folds, precluding to identify the pancreaticojejunal anastomosis (Figure 6); (2) the space between the endoscope and the pancreatic duct cannot be maintained due to the narrow intestinal lumen, resulting in blind manipulation during approach to the duct, which precludes interventional procedure in pancreatic duct (Figure 7a,b). Therefore, there have been only a few reports on the approach using BAE, and most of the studies were focused on EUS‐intervention.^31^, ^32^, ^33^, ^34^, ^35^ However, recently, novel techniques using BAE for endoscopic approaches pancreatic duct have been reported, introducing the use of a transparent long cap^36^ and gel immersion endoscopy.^37^, ^38^ Furthermore, attempts have been made using texture and color enhancement imaging.^39^, ^40^

#### Double‐balloon‐assisted ERP (DBERP) using transparent long cap–fitted endoscope (DBERP‐LC)

**FIGURE 8:**
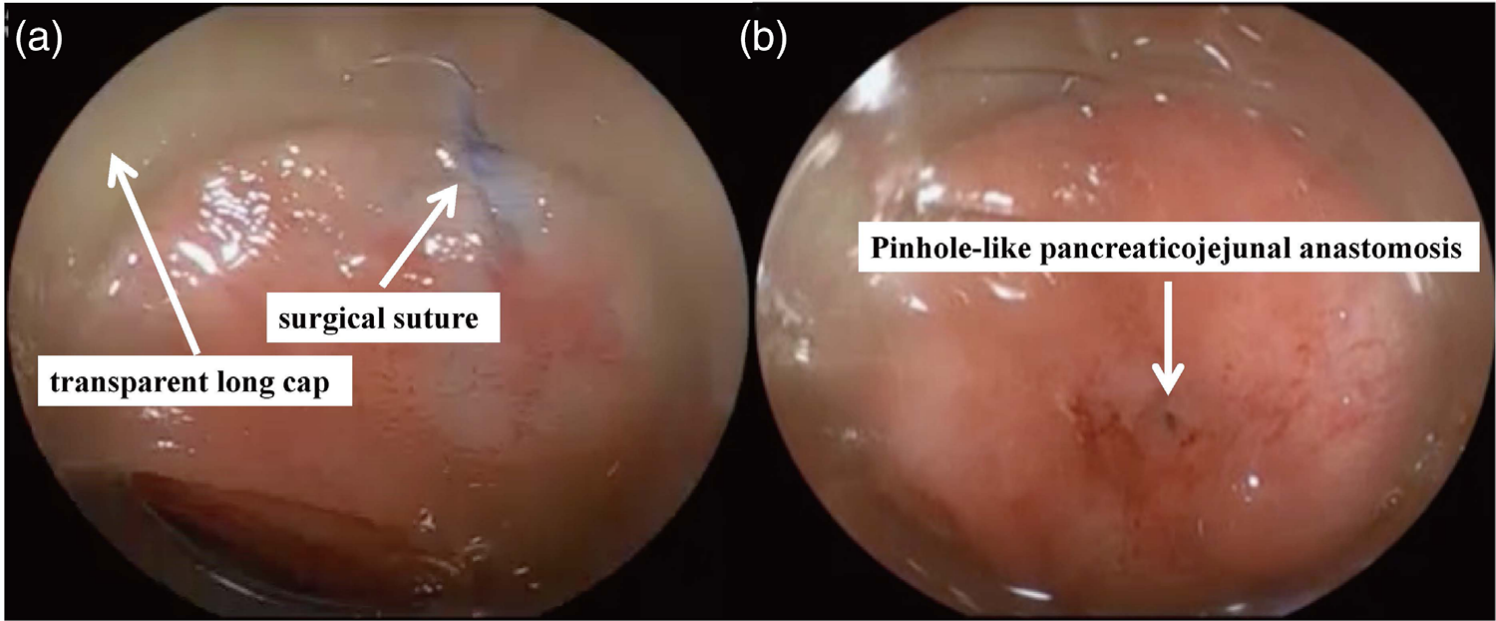
DBERC‐LC method: (a) Use a transparent long cap to locate the surgical suture that secures the pancreas to the intestinal tract, and (b) pinhole‐like pancreaticojejunal anastomosis stenosis.

The surgical suture fixing the pancreas and the intestinal tract could be a clue to identify the pancreaticojejunal anastomosis. Usually, the pancreas is fixed to the intestine at 2–3 or more points. The presence of the pancreaticojejunal anastomosis can be assumed between these surgical sutures (non‐absorbable), and it is also important to identify the ulcer scar‐like mucosa in vicinity. At this point, the use of transparent long cap is effective for several reasons. One is that pressing down the intestinal mucosa with the transparent long cap facilitates to identify the surgical suture that should contain the pancreaticojejunal anastomosis (Figure 8a). The second reason is that when carefully searching the vicinity of the surgical suture, the side of transparent long cap can flip the intestinal folds one by one to detect the ulcer scar‐like mucosa (Figure 8b). Furthermore, the transparent long cap helps maintain a distance between the endoscope and the pancreaticojejunal anastomosis, which facilitates the accurate pancreatic cannulation avoiding blind maneuver of the endoscopic interventions.^36^ Thus, the use of transparent long cap should contribute to the successful procedural achievement.

#### Gel‐immersion DBERP with long cap (GI‐DBERP‐LC)

**FIGURE 9:**
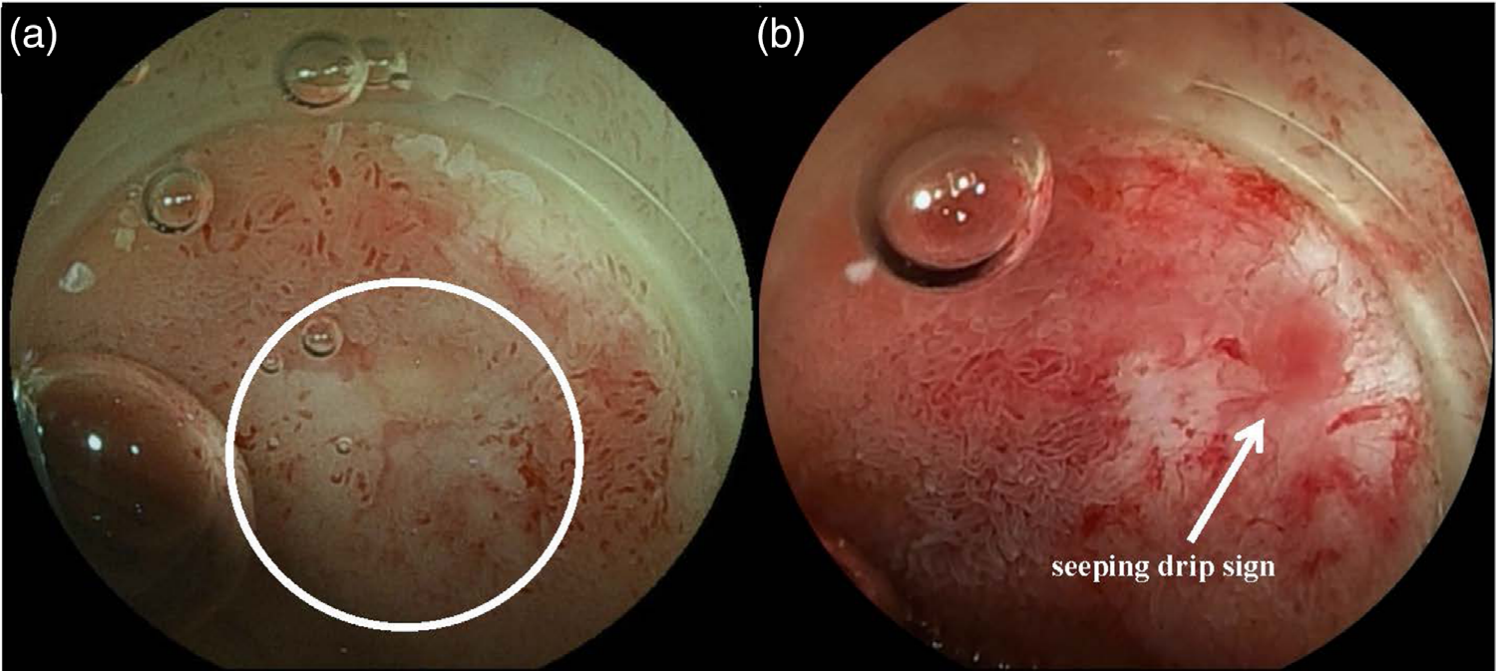
Gel‐immersion–double‐balloon‐assisted ERP with long cap (GI‐DBERP‐LC) method: (a) ulcer scar‐like lesion (inside the circle), and (b) seeping drip sign (arrow).

In cases of completely obstructed pancreaticojejunal anastomosis, which is extremely difficult to detect, DBERP‐LC may be not sufficiently effective. Thus, the gel‐immersion was applied. The gel‐immersion has the effect of opening intestinal folds as well as magnifying the endoscopic view, facilitating to capture ulcer scar‐like mucosa that should contain pancreaticojejunal anastomosis (Figure 9a). Unlike bile juice, pancreatic juice is transparent, precluding to be distinguished from intestinal fluids in the endoscopic view. However, when the gel is applied, the difference in concentration between pancreatic juice and gel allows the pancreatic juice to be visualized. By pressing down near the pancreaticojejunal anastomosis with transparent long cap, the pancreatic juice “seeping drip sign” can be seen, and the location of pancreaticojejunal anastomosis can be identified even in the case of complete occlusion (Figure 9b). Furthermore, if bleeding occurs during pancreatic cannulation under the gel, it prevents the blind maneuver, and the risk of losing pancreaticojejunal anastomosis sight is reduced, facilitating safe and accurate pancreatic cannulation and treatment intervention.^37^

## EDUCATION

Mastering the endoscopic techniques for BAE‐ERCP, including insertion into the blind end and ERCP‐related procedures, requires efforts and practice. Therefore, planning for education and training is also an imperative part of successful endoscopic procedures.

According to recent reports on the education of trainees for mastering the technique of BAE‐ERCP, first, trainees should gain experience starting with the Child (modified) procedure, in which scope insertion is considered relatively easier.^41^, ^42^ As for biliary cannulation, although there is no significant difference in success rates between approaches to the major papilla and choledocojejunal anastomotic site, the procedure time was significantly longer for the approach to the major papilla,^43^ which suggests initial performance should be for the approach to the choledocojejunal anastomotic site.

Furthermore, it has been reported that trainees with experience in colonoscopy and ERCP have higher success rates in BAE‐ERCP.^41^ BAE‐ERCP requires comprehensive mastery of various endoscopic techniques. Moreover, as BAE‐ERCP is a subtype of ERCP, it is fundamental to master ERCP, which is the basis of pancreatobiliary endoscopic treatment, and efforts should be made to master BAE‐ERCP techniques as an advanced ERCP.

Lastly, reports suggest the importance of opportunities to learn expert techniques^44^ and indicate that success rates improve with increasing experience.^15^, ^41^, ^42^, ^43^, ^44^ Therefore, enhancing the training system and mentorship at each institution is a crucial task for the future.

## TROUBLESHOOTING

### A case of difficult transpapillary biliary cannulation

**FIGURE 10:**
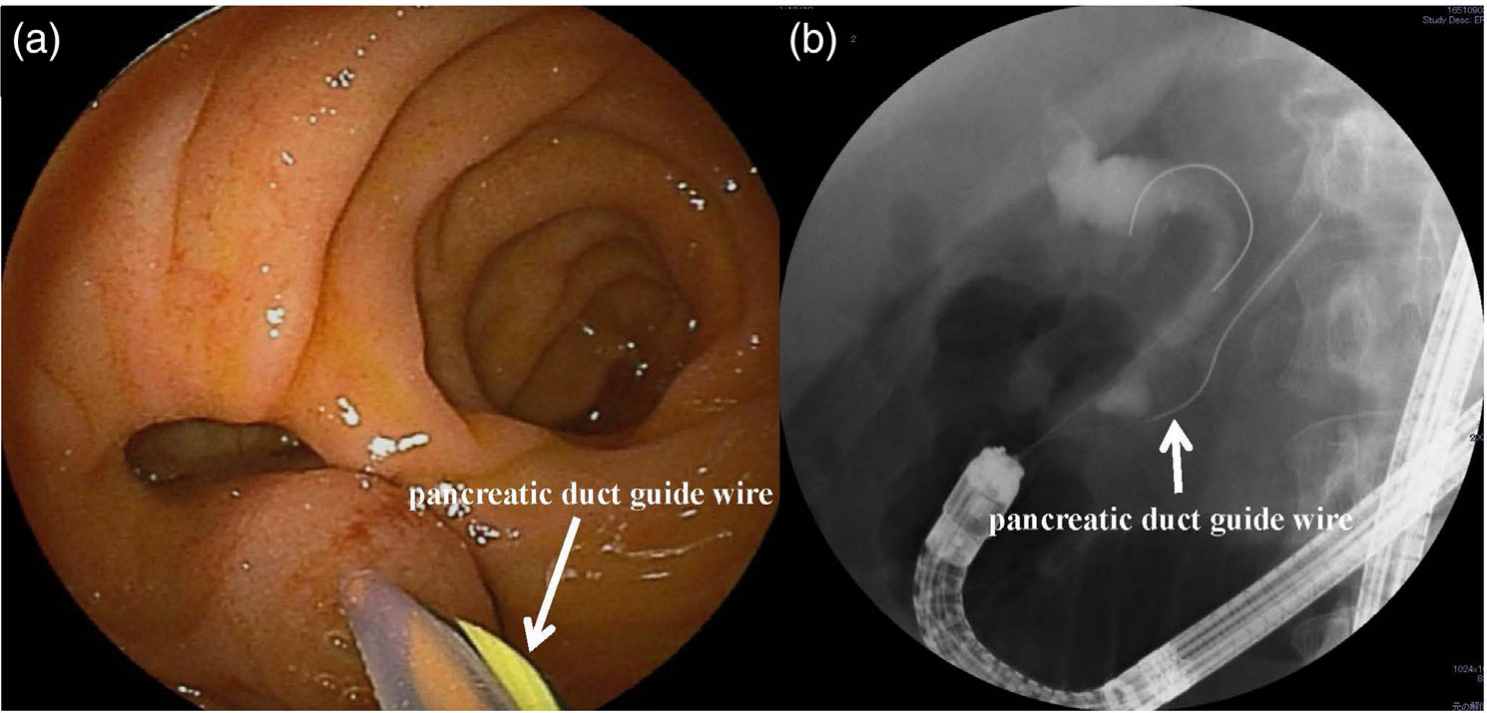
Pancreatic duct guidewire placement: (a) endoscopic image, and (b) fluoroscopic image.

As a strategy for difficult cases’ management, the pancreatic duct guide wire placement method is highly useful. By pressing guide wire, the major papilla can be positioned and fixed in the facing position. Additionally, if the main papilla is located at 6 o'clock, advancing the catheter along the upper left side of the pancreatic duct guide wire allows relatively easy biliary deep cannulation (Figure 10a,b). The utility of the pancreatic duct guide wire method using the uneven double lumen cannula (Uneven Double Lumen Cannula; PIOLAX) has been reported.^45^ However, there are difficulties when the side holes of the catheter do not face the biliary direction as intended, as the position of the side holes depends on the catheter.

**FIGURE 11:**
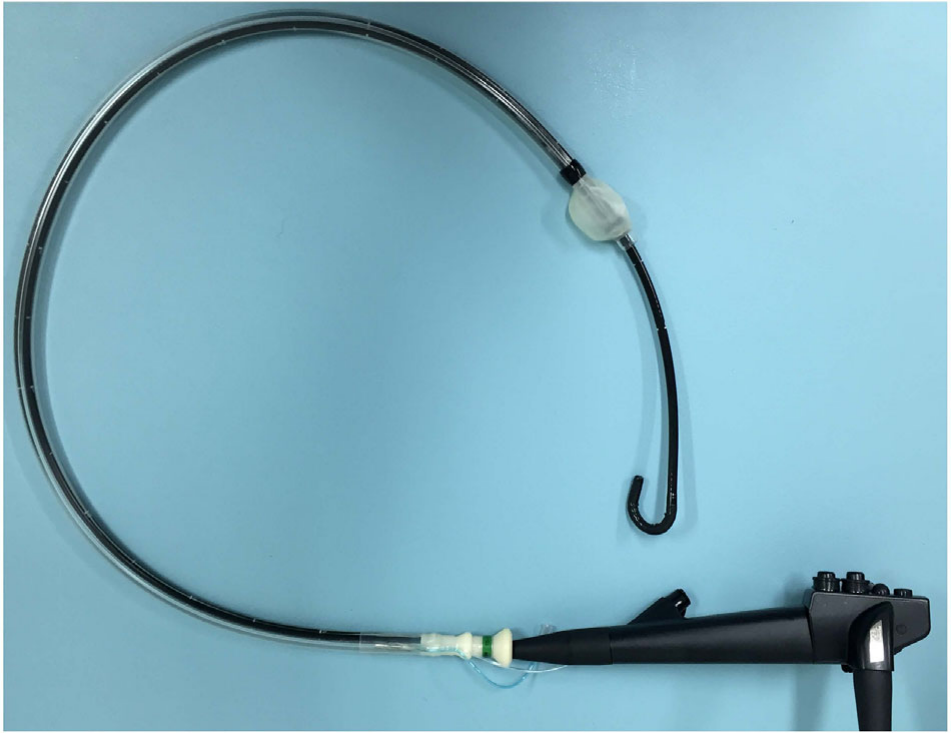
Combination method. No need to modify the overtube to exchange the long‐type ultra‐slim endoscopes.

In cases where the main papilla is difficult to visualize directly, such as when it is located from the lower duodenal angle to the horizontal portion or within a diverticulum, and when pancreatic duct imaging cannot be obtained, the combination method using a long‐type ultra‐slim endoscope with a working length of 133 cm (EC‐530XP; FUJIFILM) could be useful for biliary cannulation.^46^ Itoi et al.^47^ have also reported the usefulness of an ultra‐slim endoscope in postoperative cases. However, with a regular ultra‐slim endoscope, the working length is short, requiring the modification of the overtube and making the procedure complicated. By using the long‐type ultra‐slim endoscope, scope exchange can be performed without modifying the overtube (Figure 11), making the procedure very simple and without complexity. Therefore, it is considered a useful method as a countermeasure for challenging cases.

### A case of difficult transcholedocojejunal anastomotic biliary cannulation

One of the most challenging cases is the completely occluded choledocojejunal anastomosis. In such cases, the inability to visually recognize the anastomotic site often poses difficulties in approaching the bile duct. As countermeasures, several techniques have been reported, including the rendezvous technique combining percutaneous cholangioscopy,^35^, ^48^ interventions using EUS,^49^ and biliary puncture cholangiography using a local injection needle.^50^

## ADVERSE EVENTS

Those adverse events that occur in conventional ERCP, such as perforation, laceration, bleeding, and pancreatitis, are thought to occur in BAE‐ERCP as well. Major factors subject to adverse events are the long procedure time, B‐II, and the presence of naïve papilla.^51^ Especially, perforation is reported to occur more frequently than in conventional ERCP.^8^, ^51^ The reason for this is the presence of postoperative adhesions.^51^ However, in the case of perforation, emergency surgery can be avoided in some cases because of the adhesions, if it is handled appropriately.^52^

It is necessary to fully understand possible adverse events and to perform procedures carefully as well as to be prepared to deal with unavoidable adverse events.

## EUS‐INTERVENTION AND COMPARISON

Recently, EUS‐intervention, in which bile ducts are drained via a transintestinal approach under an ultrasound endoscope, has been accepted as a rescue therapy, particularly for cases where malignant intestinal obstruction due to peritoneal seeding physically precludes reaching the blind end with BAE. EUS‐intervention could be definitely beneficial for this group of conditions and could potentially be the primary indication.

There have been some papers reporting the more usefulness of EUS‐intervention in comparison with BAE‐interventions^53^, ^54^; however, the success rates of BAE‐ERCP in these reports are too low compared to those from other reports, necessitating further assessment. Additionally, EUS‐intervention is considered to bring about a higher risk of serious adverse events such as migrated stents into the abdominal cavity, which resulted in a fatal case.^55^, ^56^ Therefore, EUS‐intervention should not be selected without well considerations, especially for benign diseases. It is important to thoroughly understand the advantages and disadvantages of each treatment method and to carefully examine the indications for each case.

## CONCLUSIONS

Technical tips and management of difficult cases of BAE‐ERCP for pancreatobiliary disease were described along with an overview of recent studies. Significant advancements in endoscopic techniques and device development made it feasible to perform various endoscopic treatments that were previously impossible in postoperative cases. Yet there are still difficult cases to be overcome, and the completion of treatment for patients should be aimed at in combination with other rescue therapies.

## THE GUARANTOR AUTHOR OF THE MANUSCRIPT

Masaaki Shimatani

## CONFLICT OF INTEREST STATEMENT

The authors disclose no conflicts of interest.

## References

[1] Kozarek RA . The past, present, and future of endoscopic retrograde cholangiopancreatography. Gastroenterol Hepatol 2017; 13: 620–622. PMC5718180 29230140

[2] Cotton PB , Blumgart LH , Davies GT *et al*. Cannulation of papilla of vater via fiber‐duodenoscope. Assessment of retrograde cholangiopancreatography in 60 patients. Lancet 1972; 1: 53–58. 4108942 10.1016/s0140-6736(72)90059-1

[3] Takagi K , Ikeda S , Nakagawa Y *et al*. Retrograde pancreatography and cholangiography by fiber duodenoscope. Gastroenterology 1970; 59: 445–452. 5458290

[4] Forbes A , Cotton PB: ERCP and sphincterotomy after Billroth II gastrectomy. Gut 1984; 25: 971–974. 6469083 10.1136/gut.25.9.971 PMC1432487

[5] Hintze RE , Adler A , Veltzke W *et al*. Endoscopic access to the papilla of Vater for endoscopic retrograde cholangiopancreatography in patients with Billroth II or roux‐en‐Y gastrojejunostomy. Endoscopy 1997; 29: 69–73. 9101141 10.1055/s-2007-1004077

[6] Wright BE , Cass OW , Freeman ML . ERCP in patients with long‐limb Roux‐en‐Y gastrojejunostomy and intact papilla. Gastrointest Endosc 2002; 56: 225–232. 12145601 10.1016/s0016-5107(02)70182-x

[7] Elton E , Hanson BL , Qaseem T *et al*. Diagnostic and therapeutic ERCP using an enteroscope and pediatric colonoscope in long‐limb surgical bypass patients. Gastrointest Endosc 1998; 47: 62–67. 9468425 10.1016/s0016-5107(98)70300-1

[8] Shimatani M , Hatanaka H , Kogure H *et al*. Diagnostic and therapeutic endoscopic retrograde cholangiography using a short‐type double‐balloon endoscope in patients with altered gastrointestinal anatomy: A multicenter prospective study in Japan. Am J Gastroenterol 2016; 111: 1750–1758. 27670601 10.1038/ajg.2016.420

[9] Shimatani M , Mitsuyama T , Tokuhara M *et al*. Recent advances of endoscopic retrograde cholangiopancreatography using balloon assisted endoscopy for pancreaticobiliary diseases in patients with surgically altered anatomy ‐ Therapeutic strategy and management of difficult cases. Dig Endosc 2020; 33: 912–923. 32981141 10.1111/den.13848

[10] Shimatani M , Takaoka M , Tokuhara M *et al*. Review of diagnostic and therapeutic endoscopic retrograde cholangiopancreatography using several endoscopic methods in patients with surgically altered gastrointestinal anatomy. World J Gastrointest Endosc 2015; 7: 617–627. 26078830 10.4253/wjge.v7.i6.617 PMC4461936

[11] Inamdar S , Slattery E , Sejpal DV *et al*. Systematic review and meta‐analysis of single‐balloon enteroscopy‐assisted ERCP in patients with surgically altered GI anatomy. Gastrointest Endosc 2015; 82: 9–19. 25922248 10.1016/j.gie.2015.02.013

[12] Klair JS , Jayaraj M , Chandrasekar VT *et al*. ERCP with overtube‐assisted enteroscopy in patients with Roux‐en‐Y gastric bypass anatomy: A systematic review and meta‐analysis. Endoscopy 2020; 52: 824–832. 32492751 10.1055/a-1178-9741

[13] Sirin G , Hulagu S . Double balloon enteroscopy improves ERCP success in patients with modified small bowel anatomy. North Clin Istanb 2020; 7: 131–139. 32259034 10.14744/nci.2020.54533 PMC7117630

[14] Liu K , Joshi V , Saxena P *et al*. Predictors of success for double balloon‐assisted endoscopic retrograde cholangiopancreatography in patients with Roux‐en‐Y anastomosis. Dig Endosc 2017; 29: 190–197. 27637997 10.1111/den.12739

[15] Farina E , Cantù P , Cavallaro F *et al*. Effectiveness of double‐balloon enteroscopy‐assisted endoscopic retrograde cholangiopancreatography (DBE‐ERCP): A multicenter real‐world study. Dig Liver Dis 2023; 55: 394–399. 36376233 10.1016/j.dld.2022.10.014

[16] Tanisaka Y , Ryozawa S , Mizuide M *et al*. Status of single‐balloon enteroscopy‐assisted endoscopic retrograde cholangiopancreatography in patients with surgically altered anatomy: Systematic review and meta‐analysis on biliary interventions. Dig Endosc 2020; 33: 1034–1044. 33073407 10.1111/den.13878

[17] Anvari S , Lee Y , Patro N *et al*. Double‐balloon enteroscopy for diagnostic and therapeutic ERCP in patients with surgically altered gastrointestinal anatomy: A systematic review and meta‐analysis. Surg Endosc 2021; 35: 18–36. 32789590 10.1007/s00464-020-07893-x

[18] Yamamoto H , Sekine Y , Sato Y *et al*. Total enteroscopy with a nonsurgical steerable double‐balloon method. Gastrointest Endosc 2001; 53: 216–220. 11174299 10.1067/mge.2001.112181

[19] Shimatani M , Tokuhara M , Kato K *et al*. Utility of newly developed short type double balloon endoscopy for endoscopic retrograde cholangiography in postoperative patients. J Gastroenterol Hepatol 2017; 32: 1348–1354. 28019036 10.1111/jgh.13713

[20] Yamada A , Kogure H , Nakai Y *et. al*. Performance of a new short‐type double‐balloon endoscope with advanced force transmission and adaptive bending for pancreaticobiliary intervention in patients with surgically altered anatomy: A propensity‐matched analysis. Dig Endosc 2019; 31: 86–93. 30151924 10.1111/den.13261

[21] Yamauchi H , Kida M , Okuwaki K *et al*. Short‐type single balloon enteroscope for endoscopic retrograde cholangiopancreatography with altered gastrointestinal anatomy. World J Gastroenterol 2013; 19: 1728–1735. 23555161 10.3748/wjg.v19.i11.1728 PMC3607749

[22] De Koning M , Moreels TG . Comparison of double‐balloon and single‐balloon enteroscope for therapeutic endoscopic retrograde cholangiography after Roux‐en‐Y small bowel surgery. BMC Gastroenterol 2016; 16: 98. 27549034 10.1186/s12876-016-0512-6 PMC4994384

[23] Yang MJ , Kim JH , Hwang JC *et al*. Mechanistic loop resolution strategy for short‐type single‐balloon enteroscopy‐assisted endoscopic retrograde cholangiopancreatography in patients with Roux‐en‐Y reconstruction after gastrectomy (with video). Surg Endosc 2022; 36: 8690–8696. 36136178 10.1007/s00464-022-09575-2

[24] Tsutsumi K , Kato H , Muro S *et al*. ERCP using a short double‐balloon enteroscope in patients with prior pancreatoduodenectomy: Higher maneuverability supplied by the efferent‐limb route. Surg Endosc 2015; 29: 1944–1951. 25303911 10.1007/s00464-014-3889-8

[25] Tsutsumi K , Kato H , Okada H . Side‐to‐side jejunojejunostomy is favorable for scope insertion during endoscopic retrograde cholangiopancreatography in patients with Roux‐en‐Y hepaticojejunostomy. Dig Endosc 2015; 27: 708. 25959744 10.1111/den.12487

[26] Kanno Y , Ohira T , Kozakai F *et al*. Accurate endoscopic identification of the afferent limb at the Y anastomosis using the fold disruption sign after gastric resection with Roux‐en‐Y reconstruction. Dig Endosc 2022; 34: 238–243. 34516705 10.1111/den.14128

[27] Yano T , Hatanaka H , Yamamoto H *et al*. Intraluminal injection of indigo carmine facilitates identification of the afferent limb during double balloon ERCP. Endoscopy 2012; 44: E340–E341. 23012011 10.1055/s-0032-1309865

[28] Shimatani M , Takaoka M , Okazaki K . Tips for double balloon enteroscopy in patients with Roux‐en‐Y reconstruction and modified child surgery. J Hepatobiliary Pancreat Sci 2014; 21: E22–E28. 24307491 10.1002/jhbp.53

[29] Tanisaka Y , Mizuide M , Fujita A *et al*. Single‐balloon enteroscopy‐assisted endoscopic retrograde cholangiopancreatography in patients with surgically altered anatomy: A technical review. Clin Endosc 2023 10.5946/ce.2023.023 Online ahead of print. 10.5946/ce.2023.023 PMC10665628 37070202

[30] Sawas T , Storm AC , Bazerbachi F *et al*. An innovative technique using a percutaneously placed guidewire allows for higher success rate for ERCP compared to balloon enteroscopy assistance in Roux‐en‐Y gastric bypass anatomy. Surg Endosc 2020; 34: 806–813. 31139990 10.1007/s00464-019-06832-9

[31] Mizukawa S , Tsutsumi K , Kato H *et al*. Endoscopic balloon dilatation for benign hepaticojejunostomy anastomotic stricture using short double‐balloon enteroscopy in patients with a prior Whipple's procedure: A retrospective study. BMC Gastroenterol 2018; 18: 14. 29347923 10.1186/s12876-018-0742-x PMC5774028

[32] Shimatani M , Takaoka M , Ikeura T *et al*. Rendezvous technique: Double‐balloon endoscopy and SpyGlass direct visualization system in a patient with severe stenosis of a choledochojejunal anastomosis. Endoscopy 2014; 46: E275–E276. 24906098 10.1055/s-0034-1365785

[33] Ogura T , Nakamura J , Sakamoto J *et al*. Endoscopic ultrasound‐guided antegrade dilation using a drill dilator for a pancreatojejunostomy anastomotic stricture, with pancreatoscopic findings. Endoscopy 2023; 55: E617–E618. 37040880 10.1055/a-2055-1306 PMC10089794

[34] Dhir V , Isayama H , Itoi T *et al*. Endoscopic ultrasonography‐guided biliary and pancreatic duct interventions. Dig Endosc 2017; 29: 472–485. 28118509 10.1111/den.12818

[35] Chen YI , Levy MJ , Moreels TG *et al*. An international multicenter study comparing EUS‐ guided pancreatic duct drainage with enteroscopy‐assisted endoscopic retrograde pancreatography after Whipple surgery. Gastrointest Endosc 2017; 85: 170–177. 27460390 10.1016/j.gie.2016.07.031

[36] Kasai T , Shimatani M , Mitsuyama T *et al*. Endoscopic approach for a pinhole‐like benign stenosis in a pancreaticojejunal anastomosis using a double‐balloon endoscope with a clear long cap attachment. Endoscopy 2022; 54: E977–E978. 35913063 10.1055/a-1883-9514 PMC9736834

[37] Shimatani M , Mitsuyama T , Yamashina T . New treatment for completely obstructed pancreaticojejunal anastomosis using gel‐immersion double‐balloon ERP with long cap. Clin Gastroenterol Hepatol 2023; 21: A25–A26. 10.1016/j.cgh.2023.04.009 37088459

[38] Okuno N , Hara K , Haba S *et al*. Gel immersion radial incision and cutting for pancreaticojejunostomy anastomotic stricture. Endoscopy 2023; 55 (S 01): E696–E697. 37142242 10.1055/a-2058-8461 PMC10159779

[39] Toyonaga H , Kin T , Nakamura R *et al*. Successful detection of choledochojejunal and pancreaticojejunal anastomotic strictures using a novel form of texture and color enhancement imaging. Endoscopy 2022; 54: E1062–E1063. 36007914 10.1055/a-1899-8569 PMC9737425

[40] Tanisaka Y , Mizuide M , Fujita A *et al*. Use of texture and color enhancement imaging to identify the pancreatic duct orifice in a patient with a pancreaticojejunal anastomotic stricture. Endoscopy 2023; 55: E88–E89. 36216263 10.1055/a-1945-9063 PMC9829761

[41] Tanisaka Y , Mizuide M , Fujita A *et al*. Competence development of trainees performing short‐type single‐balloon enteroscopy‐assisted endoscopic retrograde cholangiopancreatography in patients with surgically altered anatomy. J Hepatobiliary Pancreat Sci 2022; 29: 1316–1326. 35594030 10.1002/jhbp.1187

[42] Uchida D , Tsutsumi K , Kato H *et al*. Potential factors affecting results of short‐type double‐balloon endoscope‐assisted endoscopic retrograde cholangiopancreatography. Dig Dis Sci 2020; 65: 1460–1470. 31562611 10.1007/s10620-019-05857-3

[43] Hosono K , Sato T , Hasegawa S *et al*. Learning curve of endoscopic retrograde cholangiopancreatography using single‐balloon enteroscopy. Dig Dis Sci 2022; 67: 2882–2890. 34973148 10.1007/s10620-021-07342-2 PMC9237007

[44] Kashani A , Abboud G , Lo SK *et al*. Double balloon enteroscopy‐assisted endoscopic retrograde cholangiopancreatography in Roux‐en‐Y gastric bypass anatomy: Expert vs. novice experience. Endosc Int Open 2018; 6: E885–E891. 29978010 10.1055/a-0599-6059 PMC6032630

[45] Takenaka M , Minaga K , Kamata K *et al*. Efficacy of a modified double‐guidewire technique using an uneven double lumen cannula (uneven method) in patients with surgically altered gastrointestinal anatomy (with video). Surg Endosc 2020; 34: 1432–1441. 31667613 10.1007/s00464-019-07228-5

[46] Shimatani M , Takaoka M , Okazaki K . Utility of endoscopic therapy using a double balloon endoscope combined with a long‐type ultra‐slim endoscope in postoperative patient allergic to contrast media (with video). Dig Endosc 2017; 29: 124–125. 10.1111/den.12736 27607757

[47] Itoi T , Sofuni A , Itokawa F *et al*. Diagnostic and therapeutic peroral direct cholangioscopy in patients with altered GI anatomy (with videos). Gastrointest Endosc 2012; 75: 441–449. 22154415 10.1016/j.gie.2011.09.038

[48] Kawakami H , Ban T , Kubota Y *et al*. Rendezvous biliary recanalization with combined percutaneous transhepatic cholangioscopy and double‐balloon endoscopy. Endoscopy 2018; 50: E146–E148. 29653447 10.1055/a-0591-2109

[49] Kin T , Hayashi T , Katanuma A . Endoscopic ultrasound‐guided fistulation between bile duct and afferent limb for treatment of complete choledochojejunal obstruction using forward‐viewing echoendoscope. Dig Endosc 2019; 31: e97–e98. 31290179 10.1111/den.13460

[50] Mitsuyama T , Shimatani M , Nagunuma M . Internal biliary drainage using double‐balloon endoscopy in patients with complete obstruction of the hepaticojejunostomy site. Dig Endosc 2021; 33: e10–e11. 33233023 10.1111/den.13868

[51] Tokuhara M , Shimatani M , Mitsuyama T *et al*. Evaluation of complications after ERCP using a short type double balloon endoscope in patients with altered gastrointestinal anatomy: A single‐center retrospective study of 1,576 procedures. J Gastroenterol Hepatol 2020; 35: 1387–1396. 32103516 10.1111/jgh.15019

[52] Vezakis A , Fragulidis G , Polydorou A . Endoscopic retrograde cholangiopancreatography‐related perforations: Diagnosis and management. World J Gastrointest Endosc 2015; 7: 1135–1141. 26468337 10.4253/wjge.v7.i14.1135 PMC4600179

[53] Bukhari M , Kowalski T , Nieto J *et al*. An international, multicenter, comparative trial of EUS‐guided gastrogastrostomy‐assisted ERCP versus enteroscopy‐assisted ERCP in patients with Roux‐en‐Y gastric bypass anatomy. Gastrointest Endosc 2018; 88: 486–494. 29730228 10.1016/j.gie.2018.04.2356

[54] Khashab MA , El Zein MH , Sharzehi K *et al*. EUS‐guided biliary drainage or enteroscopy‐assisted ERCP in patients with surgical anatomy and biliary obstruction: An international comparative study. Endosc Int Open 2016; 4: E1322–E1327. 27995197 10.1055/s-0042-110790 PMC5161123

[55] Wang K , Zhu J , Xing L *et al*. Assessment and safety of EUS‐guided biliary drainage: A systematic review and meta‐analysis. Gastrointest Endosc 2016; 83: 1218–1227. 26542374 10.1016/j.gie.2015.10.033

[56] Martins FP , Rossini LG , Ferrari AP . Migration of a covered metallic stent following endoscopic ultrasound‐guided hepaticogastrostomy: Fatal complication. Endoscopy 2010; 42: E126–E127. 20405376 10.1055/s-0029-1243911

